# Macelignan inhibits bee pathogenic fungi *Ascophaera apis* growth through HOG1 pathway

**DOI:** 10.1590/1414-431X20165313

**Published:** 2016-07-04

**Authors:** Y.K. Shin, K.Y. Kim

**Affiliations:** 1 College of Life Science, Kyung Hee University, Yongin-si, Gyeonggi-do, Korea; 2 Department of Genetic Engineering, College of Life Science and Graduate School of Biotechnology, Kyung Hee University, Yongin-si, Gyeonggi-do, Korea

**Keywords:** Bee, Ascosphaera apis, Macelignan, Antifungal agent, HOG1

## Abstract

*Ascosphaera apis* is a bee pathogen that causes bee larvae infection disease, to which treatment is not yet well investigated. The aim of this study was to investigate antifungal susceptibility *in vitro* against *A. apis* and to identify a new antifungal agent for this pathogen through minimal inhibitory concentration (MIC) assay and western blot analysis. Macelignan had 1.56 and 3.125 μg/mL MIC against *A. apis* after 24 and 48 h, respectively, exhibiting the strongest growth inhibition against *A. apis* among the tested compounds (corosolic acid, dehydrocostus lactone, loganic acid, tracheloside, fangchinoline and emodin-8-O-β-D-glucopyranoside). Furthermore, macelignan showed a narrow-ranged spectrum against various fungal strains without any mammalian cell cytotoxicity. In spite of miconazole having powerful broad-ranged anti-fungal activity including *A. apis*, it demonstrated strong cytotoxicity. Therefore, even if macelignan alone was effective as an antifungal agent to treat *A. apis*, combined treatment with miconazole was more useful to overcome toxicity, drug resistance occurrence and cost effectiveness. Finally, HOG1 was revealed as a target molecule of macelignan in the anti-*A. apis* activity by inhibiting phosphorylation using *S. cerevisiae* as a model system. Based on our results, macelignan, a food-grade antimicrobial compound, would be an effective antifungal agent against *A. apis* infection in bees.

## Introduction

Pollination is a very important process for reproduction of many crops, fruit trees, and wild plants. Among pollinators, honeybees play a major role and are the most economically valuable pollination mediators for agricultural and horticultural plants worldwide. Due to their role in nature, honeybees have a great economic impact (1).

Honey bees are continually exposed to numerous threats: pests and parasites (such as the Varroa mite or Nosema), bacterial diseases (foulbrood), fungal diseases (chalkbrood), viral diseases (invertebrate iridescent virus), and pesticides. Even if the reduction of the bee population is not fully understood, microorganism infections are one of the main reasons. Chalkbrood is a bee larvae infection disease caused by the fungus *Ascosphaera apis*. Spores of this fungus grow within the digestive track of infected bees and then develop into the mycelial form. This causes the death of the larva, which will have the appearance of chalk. The chalky larva is highly infective to the other larvae within the nest (2,3). *A. apis* can also infect adult bumble bees, which increases the potential to infect native bees via pathogen spillover (4). Except for the natural essential oils (5), no other compound that inhibits *A. apis* growth is reported.

Natural plants and medicinal herbs are a valuable resource for novel anti-microbiological agents because many have abundant biological activities and are pharmacologically safe (6-8). Natural compounds for the control of chalkbrood fungus would be a welcome alternative to synthetic fungicides. A broad range of compounds have been tested in honey bee colonies and on *A. apis* culture in an attempt to find a control for chalkbrood (2). Some of these compounds are natural plant-derived antimicrobial products (2,5,9,10). Essential oils containing citral, geraniol and citronellal were reported to have the best inhibiting effect on fungal growth *in vitro* (5). However, most herb extracts showed their best inhibitory effects at relatively low concentrations. For example, 1.5-3.5% (w/v) of *Cinnamomum cassia* and *Piper betel* extracts inhibited fungal growth (10) and more than 0.025% (w/v) of essential oils were used for chalkbrood control (5).

Since single compounds showed better efficacy and controllable activity compared with crude extract, we are focusing on highly effective single compounds for chalkbrood disease treatment. In this paper, we tried to identify a putative antifungal compound against *A. apis* among the elements from natural plants. Our medicinal herb extracts pool was screened to find new functional antifungal agents.

## Material and Methods

### Strains

*Aspergillus niger* (KACC42589), *Aspergillus clavatus* (KACC40071), *Candida parapsilosis var. parapsilosis* (KACC45480), *Rhizopus oryzae* (KACC40256) and *Saccharomyces cerevisiae* (KACC30068) were obtained from KACC (Korea Agricultural Culture Collection). *C. albicans* (KCTC7965, KCTC7270), *C. tropicalis* (KCTC7212), *C. tropocalis var. tropicalis* (KCTC17762), *C. glabrata* (KCTC7219), *Cryptococcus neoformans* (KCTC17528), and *Pichia guilliermondii* (KCTC7211) were purchased from KCTC (Korea Collection for Type Cultures). *Ascosphaera apis* was a gift from Byungsoo Yun (Kyunggi University). *S. cerevisiae* (BY4742) was purchased from Life Technologies (USA).

*A. apis* was maintained with SDA medium (11-13) at 35°C containing ampicillin (100 μg/mL) under normal atmosphere. Growing fungi were regularly monitored for contamination. Other molds and yeasts were maintained in yeast peptone dextrose (YPD) plates.

### Broth microdilution antifungal testing

For *A. apis*, the broth microdilution assay suggested by the European Committee on Antimicrobial Susceptibility Testing (EUCAST) was applied. Stock compound solutions were prepared in dimethyl sulfoxide (DMSO) with 10 mg/mL concentration. The compound was serially two-fold diluted with Sabouraud dextrose (SD; TaKaRa, Japan), Sabouraud dextrose+0.2% yeast extract (SDYE), YPD (TaKaRa) or RPMI medium (RPMI 164, Sigma, USA) without sodium bicarbonate and with L-glutamine buffered to pH 7.0 with 0.165 M morpholinopropanesulfonic acid and added with 18 g of glucose per liter to make a final concentration of 2% (11-15). One hundred microliter of the medium containing each compound ranging from 400-0.39 μg/mL were added to the 96-well flat-bottomed microtitration plates (SPL, Korea). The last well was used for sterility and growth controls. One hundred microliter of the mold inoculum suspension containing 1×10^5^ spores/mL were added to each well of microdilution plates. Plates were incubated at 35°C for 24 and 48 h. Minimum inhibitory concentration (MIC) values were defined as the lowest concentration of drug that completely inhibited cell growth.

For *aspergillus* species, the same broth microdilution assay described for *A. apis* antifungal testing was used, but with RPMI 1640 medium (14,16).

For *Candida* and *Saccharomyces* species, the broth microdilution assay recommended in the NCCLS document M27-A was used as described previously (15-18). Each compound (concentration ranged from 200-0.2 μg/mL) was serially two-fold diluted with 100 μL of RPMI 1640 medium. The diluted drug was dispensed into 96 well round bottom microdilution plates (SPL) and 100 μL of yeast inoculum was added with a concentration of 1×10^4^ cells/mL to each well of microdilution plates. Plates were incubated at 35°C for 24 and 48 h. Tests were performed at least three times.

### Antifungal synergy testing

Antifungal synergy between miconazole and macelignan against *A. apis* was tested using the broth microdilution assay and the checkerboard method (19). After miconazole and macelignan serial two-fold dilution, macelignan was dispensed into a 96-well microtitre plate with total 100 μL of drug containing SD medium per well. *A. apis* was inoculated with 100 μL of the suspension containing 1×10^5^ spores/mL.

Final drug concentrations of 12.5-0.1 μg/mL for miconazole and of 12.5-0.05 μg/mL for macelignan were obtained. MIC values of individual drugs were determined on the same plate. Tests were performed at least three times.

The fractional inhibitory concentration index (FICI) was used to determine synergistic effects of compounds using the equation: FICI=(Ac/Aa)+(Bc/Ba) where Ac and Bc are the MICs of drugs A and B in combination, and Aa and Ba are the MICs of drugs alone. FICI values ≤0.5 indicate synergy, and values 0.5-4 indicate no interaction (19).

### Cytotoxicity assays

The cytotoxicity of the test compounds was done using HepG2 cells with slight modifications from the previously reported procedure using a cell-based MTT assay (HepG2 cells were kindly provided by Prof. T.H. Yi, Kyung Hee University) (20). Cells were seeded at 1×10^4^ cells per well in 96-well plates. After 24 h of incubation, the cells were washed with PBS and cultured in Dulbecco's modified Eagle's medium (DMEM) containing various concentrations of macelignan (0-3 mM). After 48 h of incubation, MTT solution (3-(4,5-dimethyl-thiazol-2-yl)-2,5-diphenyltetrazolium Bromide, Sigma) in PBS was added to a final concentration of 0.5 mg/mL, followed by incubation for 3 h at 37°C. After, the supernatant medium was removed, and cells were suspended in 100 µL of DMSO for 10 min. Absorbance was measured at 520 nm using a micro-plate reader (VersaMax, Molecular Devices, USA). The cell proliferation rates were calculated from the optical density (OD) readings and reported as percentages of the vehicle control.

### Western blot analysis of MPK1 and HOG1 in *S. cerevisiae*

For HOG1 analysis, yeast cells (*S. cerevisiae*, BY4742; 10^8^ cells/mL) were incubated with macelignan and 0.4 M NaCl for 1 h. After disruption of the cells, 100 µg total protein was extracted and separated with SDS-PAGE. p-HOG1 and total HOG1 were detected with p-p38 (Santa Cruz, USA; sc-17852) and Hog1 antibody (Santa Cruz; sc-6815), respectively. For MPK1 analysis, cells with 10 µg/mL of macelignan were heated to 39°C to give heat stress. After 2 h, cells were disrupted and 100 µg of total protein were loaded to SDS-PAGE. p-MPK1 was detected with p42/44 MAPK (ERK1/2) antibody (Sigma) and MPK1-FLAG was detected with anti-FLAG antibody (M2; Sigma).

## Results

### Natural plant extracts

This study searched for a putative antifungal compound against *A. apis* among the elements from natural plants. First a pool of medicinal herb extracts was screened to find newly functional antifungal agents. The following nine plant extracts were identified: *Crataegus pinnatifida* (Bunge leaf), *Rhododendron brachycarpum* (fruit), the root of *Polygonum multiflorum* Thunb. (root), *Cassia brewsteri* (root), the root of *Stephania tetrandra* (root), *Gentiana scabra* Bunge (root), *carthamus tinctorius Linne* (seed), *Saussurea lappa* (root), and *Schisandra chinensis* (fruit). These extracts showed preventive effects for fungal cell growth but their effects were not strong enough (Data not shown).

### Single compound analysis for anti-*A. apis* activity

Because of the negative results from the tested herb extracts, widely known active compounds of nine herbal extracts were purchased from Chemface (China) to find an effective antifungal agent. We selected macelignan, corosolic acid, dehydrocostus lactone, loganic acid, tracheloside, fangchinoline and emodin-8-O-β-D-glucopyranoside because of their unknown antifungal function. We applied SD, SDYE, YPD or RPMI1640 medium to test antifungal activity since the standard medium for broth microdilution assay of *A. apis* was not established yet. Luckily medium effects were not detected for MIC, thereby SD medium was used for anti-fungal assay (Data not shown). Finally, macelignan showed the strongest anti-*A. apis* activity among all extracts (Table 1). Macelignan had 1.56 and 3.125 μg/mL MIC against *A. apis* after 24 and 48 h, respectively. Corosolic acid (12.5 μg/mL MIC after 24 and 48 h) and dehydrocostus lactone (50 μg/mL MIC after 24 and 48 h) had anti-*A. apis* activity but their effects were weaker than macelignan. In addition, miconazole was used as a positive control, and showed very high antifungal effects against *A. apis* (1.56 μg/mL MIC after 24 and 48 h).

**Table:**
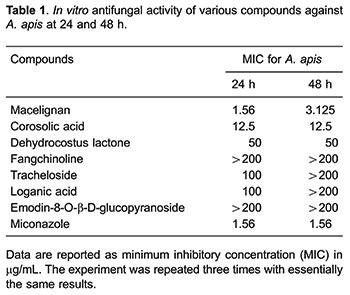

### Broad spectrum analysis

We explored whether macelignan, corosolic acid, dehydrocostus lactone and miconazole had broad spectrum against various fungus including *A. niger*, *A. clavatus*, *C. parapsilosis var. parapsilosis*, *R. oryzae*, *S. cerevisiae*, *C. albicans*, *C. tropicalis*, *C. tropocalis var. tropicalis*, *C. glabrata*, *C. neoformans* and *P. guilliermondii*. Interestingly, macelignan showed a very narrow spectrum against various tested fungus. Macelignan had anti-fungal activity specifically to *A. apis, C. neoformans* and *P. guilliermondii* (MIC = 3.125, 6.25 and 3.125 μg/mL after 24 h, respectively; Table 2). After 48 h, macelignan still showed 3.125 μg/mL MIC against *A. apis* and 6.25 μg/mL MIC against *C. neoformans,* but 200 μg/mL MIC against *P. guilliermondii* (Table 2).

**Table:**
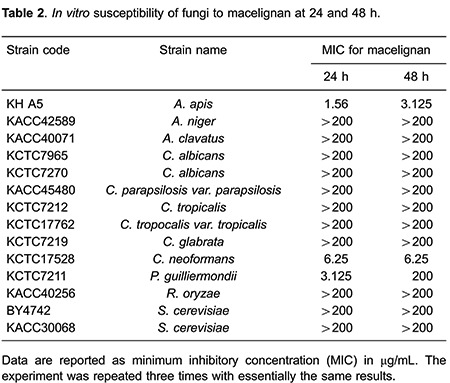

Corosolic acid showed a mild effect against *A. apis* (12.5 μg/mL MIC after 24 and 48 h, Table 3). Even if corosolic acid showed strong anti-*C. neoformans* activity (3.125 μg/mL MIC) after 24 h, MIC after 48 h was decreased to over 200 μg/mL. Unfortunately, dehydrocostus lactone did not show strong effects, but a weak antifungal activity against all of the tested fungus was observed (Table 4). MIC values for miconazole ranged from 1.56 to 25 μg/mL against all of tested fungus after 24- to 48-h incubation (Table 5).

**Table:**
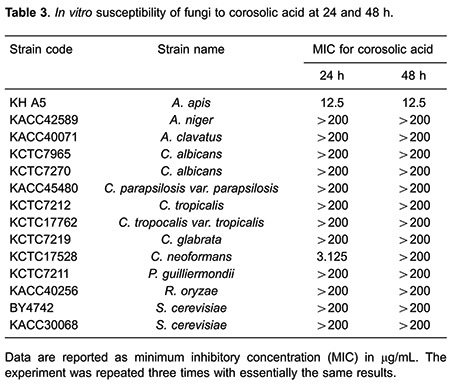

**Table:**
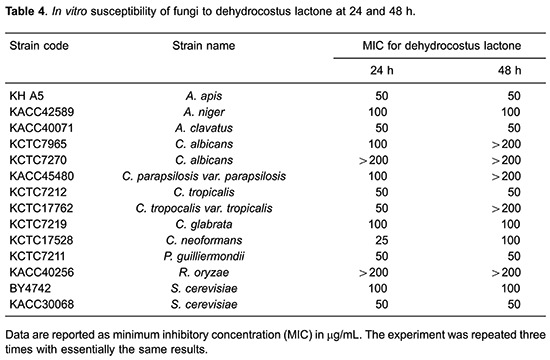

**Table:**
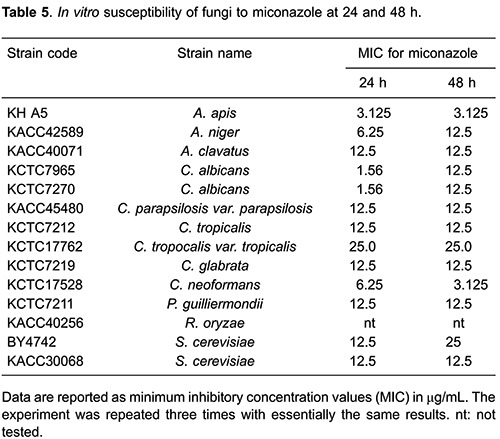

### Synergistic inhibition of *A. apis* by macelignan and miconazole

We applied the broth microdilution assay in a checkerboard method to determine if miconazole and macelignan synergistically inhibited the growth of *A. apis* (Table 6). Despite miconazole and macelignan not showing strong synergistic effects against *A. apis* (FICI=0.75 and 0.78 after 24- and 48-h incubation, respectively), miconazole MIC was reduced to 0.78 μg/mL combined with 0.39 μg/mL of macelignan. We also observed a weak growth of *A. apis* at 0.78 μg/mL miconazole with all macelignan concentrations tested and 0.78 μg/mL of macelignan with all miconazole concentrations tested.

**Table:**
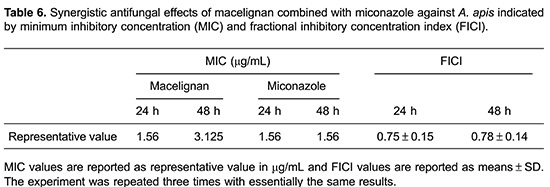

### Cytotoxicity of macelignan and miconazole

**Figure 1:**
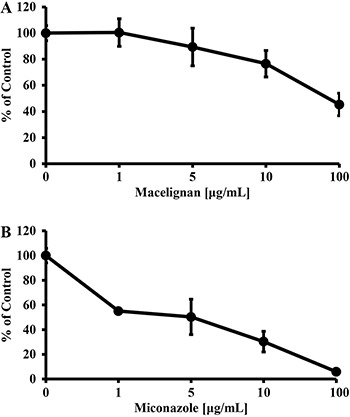
Cytotoxic effects of the macelignan (*A*) and miconazole (*B*) against hepG2 cells. HepG2 cells were treated with the indicated concentrations of macelignan and miconazole for 24 h. Data are reported as means±SD of three independent experiments.

Cytotoxicity of macelignan and miconazole against HepG2 cells was tested to dertermine whether compounds also inhibit the growth of human cells (Figure 1). Macelignan had low cytotoxicity against HepG2 cells (IC_50_=62.7±5.3 μg/mL; Figure 1A). This result is in agreement with the report that showed that macelignan had weak cytotoxicity against the HepG2 and MDA cells (21). However, miconazole had a strong cytotoxicity (IC_50_=5.9±5.5 μg/mL; Figure 1B).

### Anti-*A. apis* target validation using *S. cerevisiae*

**Figure 2:**
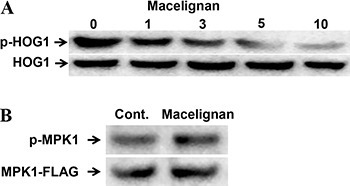
Western blot analysis of HOG1 and MPK1. *A*, *S. cerevisiae* was incubated with 1, 3, 5 and 10 µg/mL macelignan for 1 h with 0.4 M NaCl. Proteins were then extracted and detected for phosphorylated and total HOG1. Total HOG1 protein was used as a loading control (Cont.). *B*, *S. cerevisiae* with 10 µg/mL macelignan was incubated at 39°C for 2 h to activate MPK1. Proteins were then extracted and detected for phosphorylated and total MPK1. Total MPK1 protein was used as a loading control. Images are representative of at least three independent experiments.

Although *A. apis* genome sequencing was completed at 2006, translational analysis was barely performed because of poor antibody availability against *A. apis*. Therefore, in this study *S. cerevisiae* was used to validate *A. apis* antifungal target. Initially, we used the *A. apis* genome sequencing project results. We found two candidate genes *Scaffold65* and *Scaffold592*, both of which have short sequences. Compared with *S. cerevisiae* HOG1 that has 435 amino acids, predicted amino acid numbers for *Scaffold65* is 228 and for the *Scaffold592* is 94. But when we examined their sequences, we found that both are actually the same, and differences were because of the mRNA quality; moreover, there were 2 un-sequenced regions. Therefore, we prepared the genomic DNA of the *A. apis* to inspect its sequence, and generated the predicted amino acid sequence for *A. apis* HOG1 homologue. We performed an alignment with 3 different programs that showed 79.5% identity with 89.7% similarity at the conserved kinase region and 63.7% identity if the c-terminal variable region was included. Therefore, we conclude that putative *A. apis* HOG1 was very similar to *S. cerevisiae* HOG1. Total protein and phosphorylated forms of MPK and HOG1 were observed after macelignan treatment (Figure 2). After macelignan treatment, phosphorylation of HOG1 showed a dose-dependent inhibition compared with total HOG1 protein. However, MPK1 phosphorylation was not changed even with the highest concentration of macelignan treatment.

## Discussion

In the present study, macelignan exhibited the strongest growth inhibition against *A. apis* among tested compounds, followed by corosolic acid and dehydocostus lactone. Miconazole also had anti-*A. apis* activity. In addition, miconazole MIC value was reduced when combined with macelignan. Although macelignan was effective as a sole antifungal agent to treat *A. apis*, combined treatment with miconazole showed much better results.

Miconazole is an imidazole antifungal agent, which is commonly applied topically on the skin or to mucous membranes for treating fungal infections in human and veterinary medicine. It works by inhibiting the synthesis of ergosterol, a critical component of fungal cell membranes. Even though miconazole is a well-known antifungal drug and is relatively cheap, miconazole has never been reported for *A. apis* growth inhibition. In spite of miconazole having powerful anti-fungal activity, it has strong cytotoxicity (22-24). Therefore, combination of miconazole and macelignan would strengthen the cost-effectiveness of miconazole while overcoming the cytotoxicity by the usage of a natural origin compound - macelignan.

Previously developed antimicrobial compounds are usually active against various microorganisms. Overuse of broad-spectrum anti-microbial compounds facilitates the development of antibiotic-resistant infections and multi-drug resistance. Moreover, growth of probiotic organisms, which help make the microbial flora healthier, is also inhibited by anti-microbial drugs. In other words, when broad range anti-microbial agents are applied to control the pathogenic microorganism, these agents also suppress the growth of favorable organisms. Accordingly, one of the trends in the development of anti-microbial agents is to find drugs that are narrow-ranged (25). Therefore, the narrow spectrum of macelignan would be advantageous to treat a fungal disease in honey bees.

Macelignan is a natural phenolic compound derived from *Myristica fragrans* (nutmeg) (26). It possesses many biological properties, including antioxidant, anti-inflammatory, antibacterial, hepato-protective and neuro-protective activities (27-31). The molecular mechanisms for some of these biological effects have been explored in recent years, providing a foundation for understanding the pharmacological actions of macelignan (32). For example, macelignan suppressed the mitogen activated protein kinase (MAPK) signaling pathway, including p38, for the anti-inflammatory effect (33). Macelignan also protected cisplatin-induced hepato-toxicity through JNK1/2 and ERK1/2 de-phosphorylation (31) and regulated ROS-induced MAPK signaling in human skin fibroblasts (34).

MAPK cascade transmits signals from outer cell surface to the nucleus and is involved in fungal survival mechanisms against environmental stress conditions (35). MAPK signaling molecules can be good targets for antifungal drugs to avoid fungal survival against conventional drugs. Heitman's lab published research reporting that MPK1 and HOG1, MAPK pathway molecules, have antifungal drug sensitivity in *C. neoformans* (36,37). Jung and Bahn also examined stress-activated signaling pathways, including HOG and MPK1 pathways, that would be drug target components for treatment of *C. neoformans* (38). In the present study, macelignan had antifungal activity only against *C. neoformans,* other than *A. apis*, which might infer the inhibition of MPK1 and/or HOG pathways. Recently, a systems biology study confirmed MPK1 among 11 MAPK molecules as the best target for antifungal drugs (39). Therefore, inhibition of *A. apis* and *C. neoformans* growth by macelignan treatment can be assumed through regulation of MPK1 and/or HOG1 pathways. To validate which pathway was related with the anti-*A. apis* effect, *S. cerevisiae* was used for protein analysis because *A. apis* antibodies are not commercially available. When macelignan was used in MPK1 or HOG1 protein activated yeast cells, only HOG1 phosphorylation was inhibited dose-dependently. Therefore, HOG1, homolog of mammalian p38, should be a target of macelignan.

In conclusion, macelignan exhibited a strong growth inhibition against *A. apis*, with a narrow range spectrum. When macelignan was combined with miconazole, each compensated the other’s weakness, showing a synergistic effect. Macelignan, as a food-grade antimicrobial compound, would minimally influence the eco-system (40) and could be a good candidate for the treatment of bee diseases caused by microorganisms. However, macelignan is not inexpensive enough to be solely used compared with miconazole. Therefore, combined usage of macelignan and miconazole would compensate the relatively high cost of macelignan, the occurrence of antifungal-agent resistance and the cytotoxicity of miconazole. Field tests to confirm the effectiveness of macelignan will be performed, as well as the development of macelignan derivatives to increase compound stability and anti-*A. apis* activity.
